# A Practical and Scalable Tool to Find Overlaps between Sequences

**DOI:** 10.1155/2015/905261

**Published:** 2015-04-19

**Authors:** Maan Haj Rachid, Qutaibah Malluhi

**Affiliations:** KINDI Lab for Computing Research, Qatar University, P.O. Box 2713, Doha, Qatar

## Abstract

The evolution of the next generation sequencing technology increases the demand for efficient solutions, in terms of space and time, for several bioinformatics problems. This paper presents a practical and easy-to-implement solution for one of these problems, namely, the all-pairs suffix-prefix problem, using a compact prefix tree. The paper demonstrates an efficient construction of this time-efficient and space-economical tree data structure. The paper presents techniques for parallel implementations of the proposed solution. Experimental evaluation indicates superior results in terms of space and time over existing solutions. Results also show that the proposed technique is highly scalable in a parallel execution environment.

## 1. Introduction

The next generation sequencing (NGS) technology created new types of DNA sequencing challenges. The great advent of this new technology eliminates the high cost of the Sanger method. Therefore, a lab with modest equipment is currently able to sequence a modest size genome (e.g., bacterial genome). The resulting output for this technology is a group of fragments (reads), each of which is 50–1000 base pairs representing a piece of multiple copies of the genome.

This kind of output presents a challenge since these pieces should be reordered in order to obtain the complete sequence of a genome (de novo assembly). Therefore, to harvest the benefits of utilizing NGS technology, the development of space- and time-efficient algorithms to complete the assembly process has become inevitable.

All-pairs suffix-prefix (APSP) matching is one of the well-known computer science problems that has effective applications in the assembly process, especially in de novo assembly. Finding the all-pairs suffix-prefix matches can also help solve the popular shortest common superstring problem that has an important role in sequencing and mapping DNA [1]. In addition, it has applications in data compression [2].

Gusfield et al. [1] presented an optimal solution for APSP using a suffix tree [3]. It is optimal since it consumes *O*(*n* + *k* ^2^) time where *n* is the total length of all strings and *k* is the count of the strings. The suffix tree is a robust data structure that is used to solve many string matching problems. A suffix tree for a string *S* is a tree of all suffixes of *S*. Each suffix in *S* is represented by a path from the root to a leaf in the suffix tree. Despite the optimal performance of the suffix tree, it has the drawback of high memory requirements and poor locality of memory references [4].

The suffix array has been used as a substitute for suffix tree to avoid its two disadvantages [5]. A suffix array *A* of a string *S* is an array whose size is equal to the length of *S*. Each element in array *A* contains the position of a suffix in *S* where all suffixes are sorted lexicographically. Abouelhoda et al. [6] showed that any problem that can be solved using a suffix tree can also be solved using an enhanced suffix array with the same time complexity. Ohlebusch and Gog [7] presented a solution for APSP using an enhanced suffix array. This solution has the same time complexity as the solution obtained using a suffix tree, but it is faster and consumes much less space.

With the advent of next generation sequencing technology, these solutions may be considered expensive in terms of space since the required space for the text itself is *n*log⁡|Σ| bits, where |Σ| is the alphabet size and *n* is the length of text, while the space that is required to store the data structure (suffix tree or suffix array) is *n*log⁡⁡*n* bits. Compressed data structures were developed to solve bioinformatics problems using much less space with an acceptable slowdown in performance. FM index [8] is an example of such data structure that is used to solve APSP.

Dinh and Rajasekaran presented memory-efficient data structure to represent exact-match overlap graphs [9]. They mentioned that APSP can be solved using the presented data structure in *O*(*l* ^2^ *k*) where *l* is the length of one read and *k* is the number of reads assuming that all reads have the same length; otherwise *l* is a maximum length of a read. Dinh and Rajasekaran [9] used a customized compact prefix tree in the process of building the targeted data structure.

APSP has been used in the overlap stage of the genome assembly process. Two important modern assemblers are SGA [10] and Readjoiner [11]. In SGA, the FM index [8] is used to solve the problem in an indirect way as follows. The index is constructed for all strings after concatenating them in one string. Then the index is queried by the reads to find prefix-suffix matches. Other compressed versions of suffix tree and suffix array, such as RLCSA ([12–14]) and Sadakane suffix tree [15], are also used to solve APSP [16, 17].

Readjoiner is a very efficient genome assembler that, in the overlap stage, finds suffix-prefix matches with a minimal length *ℓ* by grouping all relevant suffixes in buckets. Each bucket is identified by a common prefix for all suffixes inside it. Then, after sorting suffixes inside each bucket, it finds suffix-prefix matches using the lcp-intervals concept which is introduced in [6].

This work presents a simple, efficient, space-economical, and scalable solution to APSP using a compact prefix tree. The version of prefix tree which we are using is presented as an enhancement for B-tree in [18]. This additional data structure can significantly decrease the time required to retrieve, in a set of strings *G*, the ones whose prefix is a pattern *P*. We demonstrate how to construct a compact prefix tree in Section 3. In Section 4, we explain the process of finding the longest suffix-prefix matches for each ordered pair of reads. In Section 5, we show how to parallelize our solution and describe different ways to distribute the load between threads. In Section 6, we compare our solution with previously presented solutions for APSP in terms of space and time. Finally, we present our concluding remarks in Section 7.

## 2. Preliminaries

Let Σ = {A, C, G, T} denote an ordered alphabet. A string *S* is a sequence of characters over Σ. A suffix of a string *S* is a substring of *S* that starts with a character *c* in *S* and ends with the last character of *S*, where *c* can be any character in *S*. A prefix of a string *S* is a substring of *S* that starts with the first character of *S* and ends with a character *c*, where *c* can be any character in *S*. Given two strings *S*_1_ and *S*_2_, a suffix-prefix match is a suffix of *S*_1_ which is also a prefix of *S*_2_. Finding all-pairs suffix-prefix matches (APSP) for a group of strings *G* = *S*_1_, *S*_2_, *S*_3_,…, *S*_*k*_ is finding the largest suffix-prefix match for each ordered pair of strings in *G*. In this paper, *k* denotes the number of strings and *n* denotes the total length of all strings.

## 3. Compact Prefix Tree

We define a compact prefix tree for a group of strings *G* = *S*_1_, *S*_2_, *S*_3_,…*S*_*k*_ as a tree having the following properties:

- Each string should have an identification number that represents its index in the lexicographical order of the strings in group *G*.
- Each string in *G* is represented by a path from the root to a leaf. Many strings will share partial path. If the two strings are the same, they will have the same path from the root to the leaf.
- The edge between each node *v* and its parent node *p* in the tree has a label which starts with one of the four characters (A, C, G, T).
- Each node has an interval [*r*_1_,…, *r*_2_] where *r*_1_,…, *r*_2_ are identification numbers for some strings in *G*. Since the strings are sorted, the range [*r*_1_,…, *r*_2_] represents all the strings which have a common prefix represented by a path from the root to this node.
- It is much better to store the length of a substring in the corresponding node instead of building the whole substring as a path in the tree. We will call this stored value *chain*_*len*. Each node has its own *chain*_*len* value.

**Figure 1:**
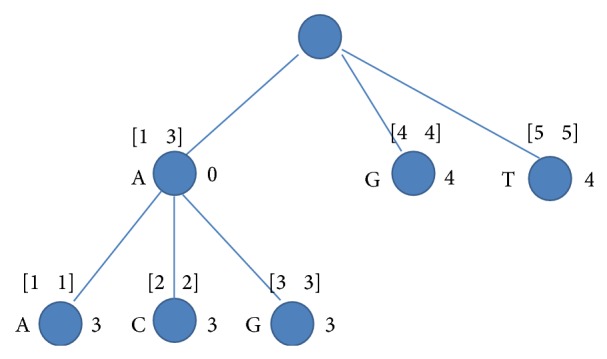
The prefix tree for strings *S*_1_ = AAGGG, *S*_2_ = ACTTT, *S*_3_ = AGGCT, *S*_4_ = GCCAC, and *S*_5_ = TCCGC.

An example for such tree is shown in Figure 1. The tree has 5 leaves since 5 strings are involved. The first child of the root has the range $\left[\begin{array}{cc} 1 & 3 \end{array}\right]$ since three strings start with character A, with *chain*_*len* = 0 since these strings differ in the second character (A, C, and G).

Accordingly, each node has at least two children since the one child case is not possible as the substring which is represented by this child would be included in the parent node.

### 3.1. Constructing the Prefix Tree

We present two methods to construct the compact prefix tree. The first constructs the tree with the assumption that the strings are sorted, while the second method constructs the tree without sorting the strings.

#### 3.1.1. Constructing the Tree after Sorting

In this method, we assume that the strings are sorted in lexicographical order. The tree construction starts with a root node. Nodes are added to the tree as the strings *S*_1_ through *S*_*k*_ are scanned in order. The first string *S*_1_ can be inserted in one step in a node. The interval for this node will clearly be [1,…, 1] where 1 is the identification number of *S*_1_. For every other string *S*_*i*_ in group *G*, where 1 ≤ *i* ≤ *k*, we match the string *S*_*i*_, character by character, with a path in the tree. Let *c* denote the current character in *S*_*i*_ to be compared with. In the matching process, the following variables are required:

- A variable *current*_*node* is used as a pointer to the current tree node.
- A variable *local*_*position* is used to indicate the position of the character *c*_1_ inside the node, where *c*_1_ is going to be compared with the current character *c* in the text. If *local*_*position* is bigger than the *chain*_*len* value of the current node, *current*_*node* will be advanced to point to the appropriate branch of current node, which is labeled by *c*.
- A variable *path*_*len* is equal to the total length of all edges in the path plus the total length of all *chain*_*len* values for all nodes in this path. This variable is important for calculating the *chain*_*len* value for new leaves.
- To find exactly what character *local*_*position* is pointing to, we find the character in the position [*Path*_*len* + *local*_*position*] of the original text.

A match may occur in two cases:

- *local*_*position* is less than or equal to *chain*_*len* of the current node, and then we are still within the same node and *c* matches the character which *local*_*position* is pointing to.
- Otherwise, a match occurs if there is a branch for the current node, labeled by *c*. In this case, *current*_*node* should point to that branch node which becomes now the current node. An update to the interval of this current node should be done by simply changing the upper bound of its interval to *i*.

A mismatch may occur in two cases:

- It occurs when comparing *c* within a node; that is, *local*_*position* is less than or equal to *chain*_*len* of the current node. In this case, the following steps should be executed in order:
  - The current node *v* should be split into two nodes *v*1 and *v*2 where *v*1 is the parent of *v*2.
  - *chain*_*len* of *v*1 becomes equal to *local*_*position*-1 and the interval for *v*1 becomes the same as *v*.
  - *chain*_*len* of *v*2 becomes equal to *chain*_*len* of *v*1 − *local*_*position*. The lower bound of *v*2 becomes the same as *v*.
  - The upper bound of *v*2 becomes equal to the upper bound of *v* − 1.
  - Create a new node *v*3 which will be the new branch of *v*1, labeled with *c*. *chain*_*len* of *v*3 becomes equal to the length of *S*_*i*_ − *path*_*len* and the interval for *v*3 is [*i*,…, *i*].
- It occurs when *local*_*position* is greater than *chain*_*len* of the current node, and then *current*_*node* should point to the appropriate branch which is labeled by *c*. If no such branch exists, then we have a mismatch. In this case we just create a node with a range of [*i*,…, *i*] and a *chain*_*len* which is equal to length of *S*_*i*_ − *path*_*len*.

**Figure 2:**
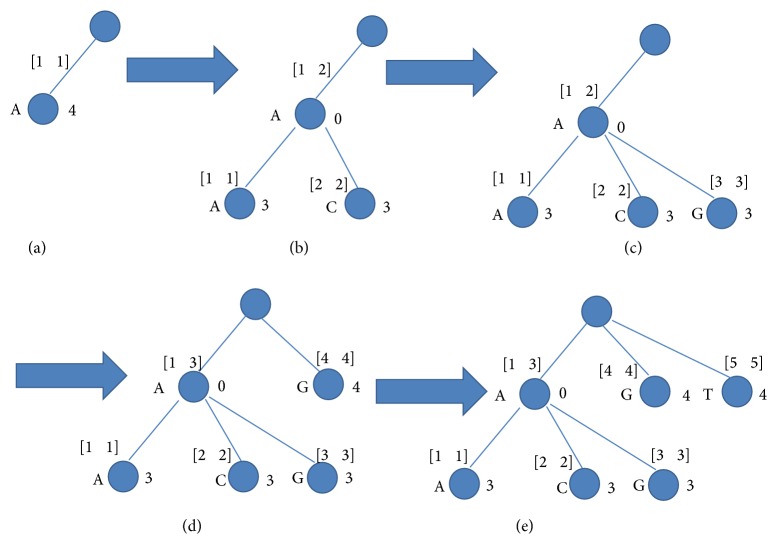
Construction of prefix compact tree after sorting the strings. Each stage represents the current tree; the strings are *S*_1_ = AAGGG, *S*_2_ = ACTTT, *S*_3_ = AGGCT, *S*_4_ = GCCAC, and *S*_5_ = TCCGC.

Figure 2 demonstrates the stages of constructing the tree. The character which is on the left side of a node is the label of the node. The interval above the node denotes its string interval. The number shown on the right side of the node denotes *chain*_*len* of the node.  Algorithm 1 demonstrates the pseudocode for constructing the tree.

The following refers to the example in Figure 2. The line numbers refer to the pseudocode illustrated in Algorithm 1. The first string *S*_1_ is inserted in one step (Figure 2(a)); the branch A for the root is created with an interval [1,…, 1] and *chain*_*len* = 4 which is the length of *S*_1_ excluding the first character A which represents the branch (lines 26–29). Considering the second string *S*_2_, we have a match with the first character (lines 6–8), while the second character “C” causes a mismatch. Since the mismatch occurs when *local*_*position* is still less than or equal to the *chain*_*len* of the current node (mismatch inside the node), we split the current node *v* into two nodes: *v*1 with *chain*_*len* = 0 and an interval [1,…, 2] and *v*2 with *chain*_*len* = 3 and an interval [1,…, 1]. Then we add *v*3 as a branch to *v*1 with *chain*_*len* = 3 and an interval [2,…, 2] (lines 9–19) (Figure 2(b)). Regarding the third string *S*_3_, we have a match with the first character (lines 6–8) and a mismatch with the second character. Since *local*_*position* > *chain*_*len* of the current node, and the current node does not have a “G” branch, we simply add a node with *chain*_*len* = 3 and an interval [3,…, 3] (lines 27–30) (Figure 2(c)). Both strings *S*_4_ and *S*_5_ get a mismatch with the first character. Therefore, they are inserted directly in one step each (lines 26–29) (Figures 2(d) and 2(e)).

Since processing each character in every string is done in constant time, the tree can be constructed in *O*(*n*) time. Since sorting the strings consumes also *O*(*n*) time using radix sorting, the time complexity stands. Since each internal node has at least two children, and the number of leaves in the tree is *k*, the number of internal nodes is at most *k* − 1. Accordingly, the tree can be constructed using *O*(*k*) space. Therefore, the space requirement of the solution is determined by the space needed to store the text, which is *O*(*n*log⁡|Σ|) bits since *n* is much bigger than *k*.

#### 3.1.2. Constructing the Tree without Sorting

This section presents a method for constructing the prefix tree without the need for sorting the strings. This method has two stages:

- Constructing the tree with no consideration for the intervals.
- Traversing the tree in a depth-first search fashion and updating the intervals.

In the first stage, we use the same construction method which is used for the sorted input in the previous section but with one difference: we ignore the intervals for internal nodes since the identification numbers for the strings are not known. For leaves, we use the current index of the string in the list of unordered input strings as their identification numbers (e.g., for *S*_3_, the interval [3,…, 3] is used).

In the second stage, a depth-first traversal for the constructed tree is required to update the intervals. The intervals of each internal node are updated after updating the intervals of its children. A counter is used to assign identification numbers for leaves. When a leaf is visited, the current value of the counter is assigned to it and the counter is incremented. For example, in Figure 2, the interval of the first branch of the root will be updated to be [1,…, 3] after updating the leaves with the intervals [1,…, 1], [2,…, 2], and [3,…, 3]. There is one case that should be considered: when two strings are exactly the same, they will have the same exact path in the tree. Since we ignore intervals during the insertion stage, there will be no way to distinguish one of these strings from another (here all strings are kept as input for the problem; in fact such a string is typically filtered out in a genome assembler). This issue can be handled using k lists in the insertion stage; for each string *S*_*i*_, we add *i* into the list of *S*_*j*_ if *S*_*i*_ and *S*_*j*_ are exactly matched, assuming *S*_*j*_ is processed before *S*_*i*_. These lists are used later by assigning a new consecutive identification number for each string. Clearly, strings in the same list will have sequential numbers. The pseudocode for the traversal stage is shown in Algorithm 2.

## 4. Finding All-Pairs Suffix-Prefix

Both methods in Section 3 produce the same output, which is an efficient prefix tree to be used to solve APSP. In this section, we present an effective technique for finding a solution for APSP.

In this method, every suffix in every string is tested, starting from the largest proper suffix (i.e, the suffix which starts at position 2). If a suffix *f* in string *S*_*i*_ matches a path in the tree (there is a path which starts with the root and ends in a node with a range [*r*_*d*_,…, *r*_*x*_] in the tree), then *f* represents the longest suffix-prefix match between *S*_*i*_ and every string included in the range [*r*_*d*_,…, *r*_*x*_]. For each string, every suffix should be processed. Accordingly, processing the suffixes of each string consumes *O*(*l* ^2^) time where *l* is the maximal length of a string (which is typically less than 1000 in the genome assembly context). Therefore, the time complexity for this method is *O*(*kl* ^2^) where *k* is the number of strings.

We write *Su*_*ij*_ to denote the suffix *j* of the string *i*. For each string *S*_*i*_ in group *G*, we check if the current suffix *Su*_*ij*_ exactly matches a path in the tree where 2 ≤ *j* ≤ *l* and *l* is the length of *S*_*i*_. Let *v* denote the current character in *Su*_*ij*_. We distinguish three cases:

- We reached the last character in *Su*_*ij*_, which means that *Su*_*ij*_ exactly matches a path in the tree. In this case, *j* will be the starting position for the longest suffix-prefix match between *S*_*i*_ and every string included in the interval of the current node.
- If *local*_*position* is less than or equal to *chain*_*len* of the current node, then we are still within the same node and the current character *v* in suffix *Su*_*ij*_ either matches the character which *local*_*position* is pointing to or does not match it and accordingly *Su*_*ij*_ does not represent any suffix-prefix match.
- If *local*_*position* is greater than the *chain*_*len* of the current node, then *current*_*node* should point to the appropriate branch which is labeled by *c*, where *c* is the current character to be compared in *Su*_*ij*_. If no such branch exists, then *Su*_*ij*_ does not represent any suffix-prefix match.

This method is easy to implement. The pseudocode is shown in Algorithm 3. The variables *Path*_*len*, *local*_*position*, and *current*_*node* defined in Section 3.1 are used in this algorithm.

### 4.1. Prefiltering the Reads

In our previous discussion, we used the *k* original strings (reads) as an input for our overlap solution; therefore, the size of the output is *k* ^2^. However, in the context of genome assembly, some filtration is applied on the *k* input reads and some redundant reads are removed before finding the overlap. Our solution can easily and efficiently perform such filtration:

- If a string *S*_1_ matches a prefix of another string *S*_2_, then *S*_1_ can be removed. The removal procedure can be handled in the construction process. We have 2 cases:
  - *S*_2_ is processed first when constructing the prefix tree. In this case, *S*_1_ will match a path in the prefix tree and it is simply removed. This case is possible only when construction is done with no sorting.
  - *S*_1_ is processed first when constructing the prefix tree. If the strings are sorted, then we assume that sorting will filter *S*_1_. Otherwise (with no sorting case), processing *S*_1_ will reach the leaf which has the interval [*S*_1_# *S*_1_#] where *S*_1_# is the identification number of *S*_1_, and there is no need for executing the procedure in Algorithm 2. Instead, the interval of the leaf will be updated to *S*_2_ identification number.
- If a string *S*_1_ matches a suffix *Su* of another string *S*_2_, then *Su* will match a path in the prefix tree ending with a leaf. In this case, *S*_1_ should be removed. A vector of *k* bits can be used to indicate if a string is removed.

## 5. Parallelizing the Algorithm

In this section, we show how to parallelize the tree construction, and then we explain different techniques to parallelize the solution. In our discussion, we assume the availability of a shared memory multicore computer.

### 5.1. Parallelizing the Construction of Prefix Tree

A quick and fast way to parallelize our solution is to let each processor work on strings which start with a specific character in the alphabet (A,C,G,T). For example, processor 1 constructs the part of tree that corresponds to strings that start with “A”. Processors 2, 3, and 4 construct the parts of the tree corresponding to the strings starting with C, G, and T, respectively.

The concept can easily be extended to more than 4 processors. For 16 processors, as an example, the load can be distributed based on the first two characters. In other words, the tree construction is distributed such that each processor is responsible for processing strings starting with the prefix XY, where X and Y are characters in the alphabet. Accordingly, processor 1 works on strings starting with “AA”, processor 2 works on strings starting with “AC”, and so on. The advantage of this method is the absence of any communication between processors.

### 5.2. Parallelizing Finding the APSP Matches

In this section, we show several techniques to parallelize our solution. The first direct technique simply divides the strings among processors so each processor gets equal number of strings. This method requires almost no modification to the sequential version of the algorithm and it scales very well. The problem is that it does not acknowledge the differences in length between the strings which may decrease the efficiency because of load imbalance.

Another way to parallelize the solution is to estimate the required load and therefore the amount of work that each processor should optimally have. Then, strings are assigned one by one to a processor until the load exceeds its estimated share. Since processing each string requires processing every suffix in it, the total amount of work (number of comparisons) for each string can be estimated as follows:

(1) $$\begin{array}{c} W_{S}=\frac{\left(\left|S\right|\times \left(\left|S\right|+1\right)\right)}{2}, \end{array}$$

where *W*_*S*_ is the required work for processing *S* and |*S*| is the length of *S*. Accordingly, a processor's optimal share can be estimated as follows:

(2) $$\begin{array}{c} \text{Processor's}\;\;\text{optimal}\;\;\text{share}=\frac{\sum_{i=1}^{k}l_{i}}{p}, \end{array}$$

where *p* is the number of processors. An array, *start*_*p*, with the size of *p* is used, where *p* is the number of used processors (threads). It contains the number of the first string to be processed by a processor.

To illustrate the concept, a simple example is shown. Let G = {ACC, AATC, CGTC, TTA, TGA, CCAT} be a group of strings that 3 processors are working on. The number of steps to process these strings is 6, 10, 10, 6, 6, and 10. Accordingly, the share for each processor is 16. Processor 1 gets strings 1 and 2, processor 2 gets strings 3 and 4, and processor 3 gets strings 5 and 6. However, this may not be the case in practice. The pseudocode is shown in Algorithm 4.

A third technique which may be used to parallelize the solution is to assign an initial load, which is a range of strings, for each processor. A shared pointer is used to indicate the starting point of a new range for a free processor. When a processor finishes executing its initial load, it gets a new range of strings using the shared pointer, then it updates the value of the shared pointer. This technique requires mutual exclusion for updating the shared pointer.

The solution can also be parallelized (fourth technique) using a greedy algorithm. An array *r* of size *k* (number of strings) is used where *r*[*i*] is the processor number which is going to handle string *i*. Another array *y* with the size *p* (number of processors) is also used to maintain the current shares for processors. For every string *S*, *S* is assigned to a processor *p*1 which has the least share (i.e., *r*[*S*] = *p*1, *y*[*p*1] = *y*[*p*1] + *W*_*S*_).

Notice that the granularity of load distribution over processors is the string. In other words, a single string is not processed on more than one processor. For a small number of strings, this may be a problem. For example, consider one huge string *S*. The performance will be limited by the time for processing this string on one processor. However, in practice, this should not be a problem since *k* is much bigger than *ℓ* where *k* is the number of strings, and *ℓ* is the maximum length of a sequence.

## 6. Experimental Evaluation

In this section, we evaluate the performance of our solution and its scalability in a parallel execution environment.

### 6.1. Experimental Setup

Our solution has been implemented in C++. We use String Overlap Finder (SOF) to refer to this solution, which is available for download from http://confluence.qu.edu.qa/download/attachments/9240580/Prefix.tgz.

In this section we compare SOF with several previously presented solutions for APSP: suffix tree, enhanced suffix array, Sadakane suffix tree, and the overlap stage of SGA and Readjoiner (version 1.2).

These solutions have been downloaded from the sources shown in Table 1.

LEAP is another efficient genome assembler which is implemented in [9]. We did not use LEAP in our comparisons since LEAP, unlike Readjoiner and SGA, does not offer a seperate stage for finding overlaps which makes estimating the time required for finding overlap matches out of the overall time very hard. Nevertheless, it has been demonstrated in [11] that Readjoiner has better time and space consumption than LEAP.

We use the OpenMP flag to support multithreading. The program takes few optional parameters: sorting option, minimal length for a suffix-prefix match, number of threads, type of output, and load distribution. The sorting option enables or disables sorting before constructing the tree. We distinguish three different types of output: outputting all matches, outputting only maximum matches using a two-dimensional array, and no output (the results are not printed or stored in any data structure).

Our results are obtained using the following options for SOF: no sorting, dividing strings equally between threads, and output = 2 (which means outputting all suffix-prefix matches, not only the longest), except for the large data sets where output = 0 (no output) option is used (the size of output is *O*(*k* ^2^)). Since many of our samples have a small read length, the minimal match length which is used is 30 unless another value is mentioned.

We used two types of data sets: random and real. The random data are generated by a program that outputs random *k* strings with random lengths but with a total length of *n* where *n* and *k* are specified by the user. The random values were drawn from a uniform distribution.

As with most other solutions like Readjoiner, the input file is encoded in SOF using fixed-length encoding. This step lowers our space requirement dramatically but increases the processing time, only when a small number of reads are used.

The real data are the complete EST database of* C*.* elegans* which is downloaded from http://www.uni-ulm.de/in/theo/research/seqana. We also obtained three complete EST databases of* Citrus clementina*,* Citrus sinensis*, and* Citrus trifoliata* from http://www.citrusgenomedb.org/ and the complete EST database of* Atta cephalotes* from antgenomes.org. We also obtained 4 large real data sets from NCBI website.  Table 2 shows our data sets.

Tests are performed in two environments:

- A space-limited environment, which is a modest machine: Linux Ubuntu version 11.10, 32-bit with 3 GB RAM, Intel 2.67 GHZ CPU with 4 cores, and 250 GB hard disk. We refer to it as machine A. It is used to evaluate SOF time and space requirements when limited resources are available.
- An AWS instance with 16 cores to evaluate the parallelization of our solution and compare SOF with Readjoiner. We refer to it as machine B. It is used to evaluate SOF time and space requirements when dealing with large data sets.

### 6.2. Experimental Results

#### 6.2.1. Evaluating SOF with Limited Resources (Machine A)

**Figure 3:**
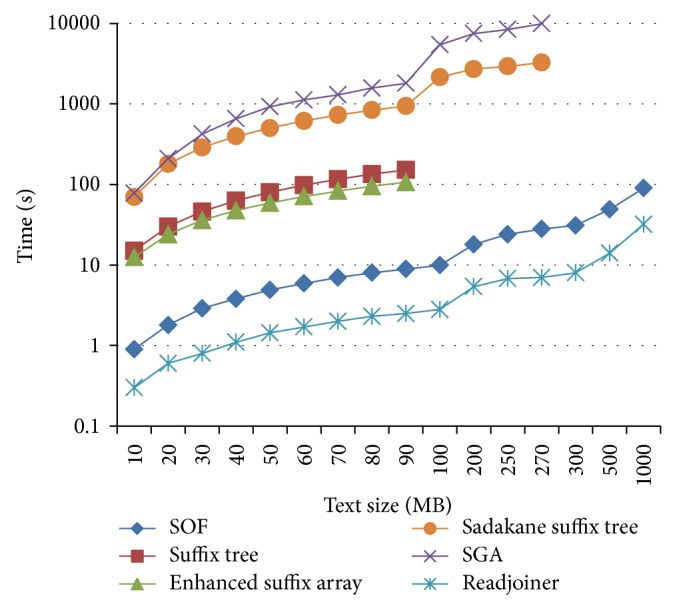
Time comparison between six different solutions for APSP, running on machine A, with random data. Logarithmic scale is used. SGA, Readjoiner, Sadakane suffix tree, and SOF are tested with a minimal match length = 15, while other solutions are tested with minimal match length = 1 (which is the only option).

The time required for all 6 solutions, running on machine A (the modest machine), is shown in Figure 3. Since not all solutions support multithreading, we evaluate and compare their performance on a single processor machine. SGA and Readjoiner do not perform well with minimal match which is less than 10, so we test all solutions, except suffix tree and enhanced suffix array (the only choice for both regarding minimal match is 1), with minimal match length = 15. Due to high space consumption, we could not run the suffix tree and enhanced suffix array with more than 90 MB. Our results show clearly that Readjoiner and SOF outperform other solutions. The advantage for Readjoiner over our solution is surmounted by the fact that we ignore the time for prefiltering, which is a prerequisite for the overlap stage in Readjoiner but is not needed for SOF.

**Figure 4:**
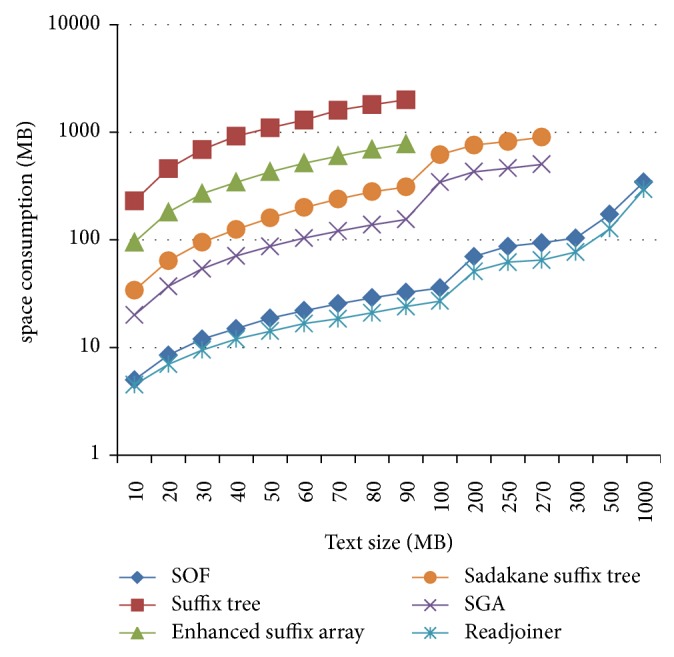
Space comparison between six different solutions, running on machine A, with random data.

The space requirements for these tests are shown in Figure 4. Clearly, Readjoiner and SOF are the most effective in terms of space.

The shown results are expected. SGA and Sadakane suffix tree use compressed full-text data structures. To build them, a considerable construction time is required, *O*(*n*log⁡⁡*n*) in the worst case. In addition, the time required for some operations, such as the *locate* operation, may not be constant, which is the case in a standard suffix tree/array. On the other side, the overlap technique in SGA is not optimal, unlike the case for suffix tree/array which requires *O*(*n*) time. SGA has a good space consumption, but it is still at least *O*(*n*log⁡⁡Σ) with a higher constant than both SOF (*O*(*k*) for constructing a prefix tree and *O*(*n*log⁡⁡Σ) for storing the text) and Readjoiner.

**Figure 5:**
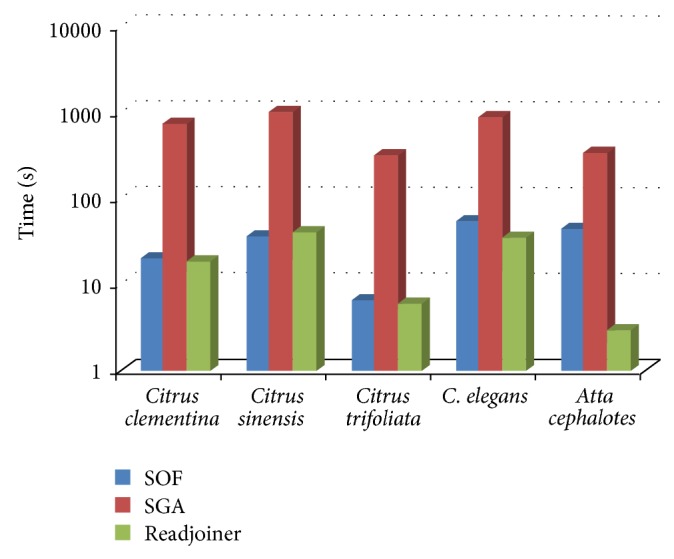
Time comparison between 3 different solutions, running on machine A, with real data using 4 threads.

**Figure 6:**
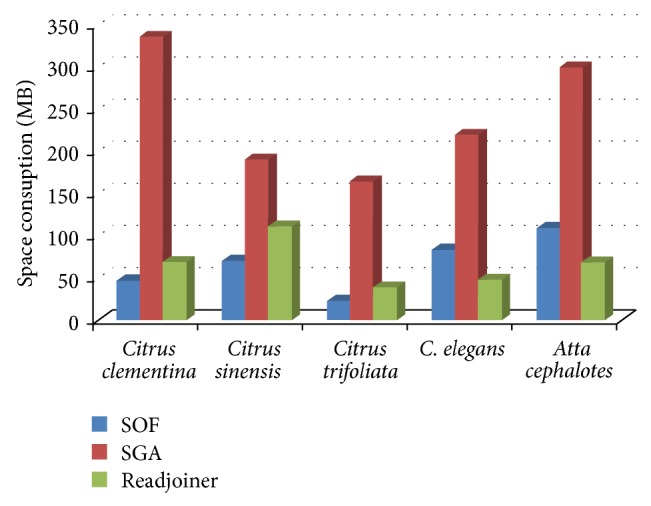
Space comparison between 3 different solutions, running on machine A, with real data.

Using real data, SGA, Readjoiner, and SOF were tested on machine A using 4 threads (the maximum number of threads on machine A). We ignore other solutions since they do not support multithreading or they are remarkably slow. The time and space consumptions are shown in Figures 5 and 6. SOF had the best performance when using multithreading in most cases. In these results, the prefiltering time for Readjoiner is ignored. Both SOF and Readjoiner performed much better than SGA. We attribute the impressive performance and low space requirement of Readjoiner when testing with* Atta cephalotes* to the low number of strings in this data set. This is due to the fact that Readjoiner finds distinct prefixes which can be candidate for suffix-prefix matches. This procedure is related to the number of strings in the data set.

#### 6.2.2. Evaluating SOF with Large Data Sets (Machine B)

**Figure 7:**
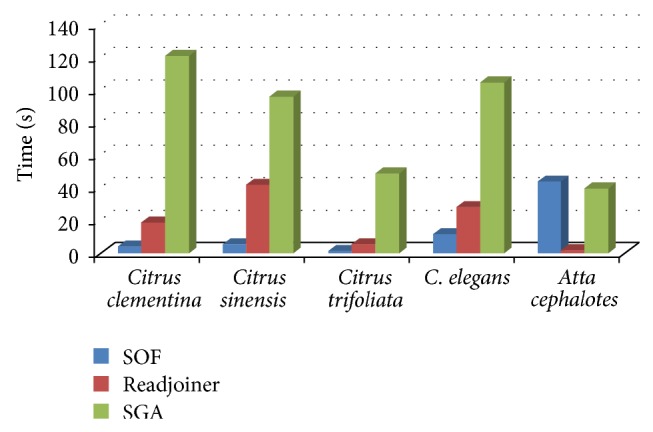
Time comparison between SOF and Readjoiner, using 16-core AWS machine with real data. We could not run Readjoiner with 3 out of 5 data sets using all 16 threads, so we demonstrate the results using the maximum number of threads which Readjoiner can use.

The parallelization of SOF and its performance on real and large data sets with large numbers of strings are evaluated and compared with Readjoiner and SGA using machine B (an AWS 16-core node). Results for real data are shown in Figure 7. While we were able to run SOF using 16 threads with all data sets, we could not run Readjoiner with any of* Citrus* data sets using more than 10 threads. Unlike SOF whose space requirement does not change with the number of threads, the space requirements for Readjoiner increase as the number of threads increases.  Table 3 shows the space consumption of Readjoiner and SOF using different numbers of threads. In the first sample (*Citrus clementina*), for example, the space consumption for Readjoiner increases more than 100% when 4 threads are used and more than 250% when 9 threads are used. As a result, the space consumption of Readjoiner exceeds that of SOF for large number of threads. Unfortunately, the error which occurs when running Readjoiner with more threads prevented us from showing even a higher difference in space consumption.  Table 4 shows the time consumption for SOF and Readjoiner. SOF demonstrates better scalability in most cases.

Readjoiner finds overlaps in several steps. In each step, it uses buffers in order to prepare the output for the next step. When the data in a buffer is processed, the buffer is refilled again and a new chunk of data is processed. This is repeated until the whole set of data is processed. In a multithreading environment, these buffers are most probably created for each thread in order to process multiple chunks at the same time, which may explain the increase in the space consumption when more threads are used.

The results for testing SOF using large data sets with large numbers of strings are shown in Table 5. These datasets are equal to or bigger than the ones which are tested with LEAP [9] in terms of size and number of strings.

We excluded LEAP in our comparison since LEAP does not offer the ability to investigate each stage of the assembly process separately. Therefore, we cannot single out the performance of the relevant overlap stage. However, we tested LEAP's ability to handle our datasets. The program receives a signal 11 as an indication for a segmentation fault when running with datasets 1, 2, and 4 from Table 4 and SRR500004 from Table 5. However, it finishes executing when running with other datasets but with very long times (3.5 hours for SRR866986 and more than 17 hours for SRR098909).

We could not run Readjoiner with any of the data sets in Table 5. For example, we received an “assertion failed” message when running Readjoiner with SRR500004. The prefiltering stage shows a segmentation fault when running Readjoiner with ERR1257766, SRR866986, and SRR098909. Accordingly, the overlap stage is not reached with these data sets. We received the same messages in single- and multicore environments. Other solutions (Sadakane suffix tree and SGA) consume a large amount of time (more than 6 hours for the 10 GB file).

## 7. Conclusion

Both Readjoiner and SOF are fast and space-economical techniques for solving APSP when compared to other solutions. Despite the advantage for Readjoiner in terms of space and time when no multithreading is used, SOF is simple and easy to implement and performs well on a simple machine. In addition, on multicore and parallel machines, SOF exhibits better performance and scalability as compared to Readjoiner. Unlike SOF, Readjoiner's space consumption increases when using more threads. As a result, SOF can consume less space and time than Readjoiner when both are using multithreading. SOF can also be efficiently used with huge data sets and large numbers of strings beyond the problem sizes and number of strings that Readjoiner can support.

## Figures and Tables

**Algorithm 1:**
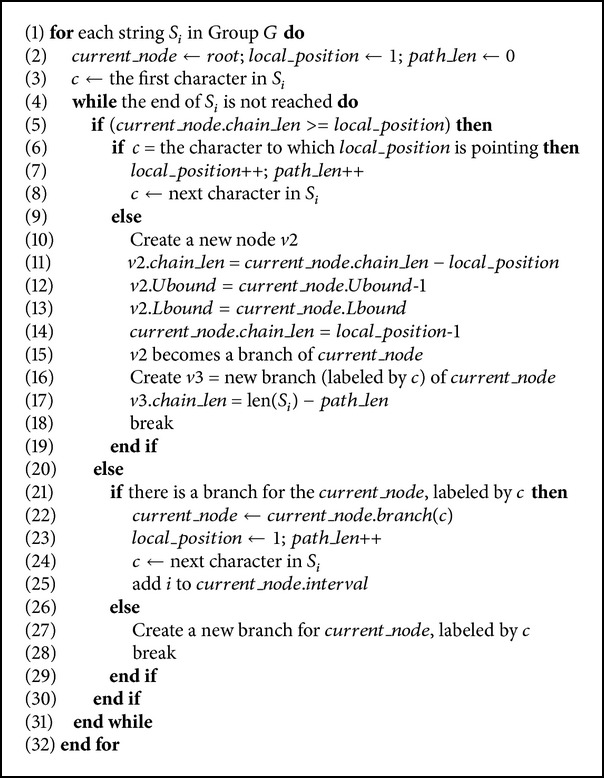
Constructing the tree after sorting the sequences.

**Algorithm 2:**
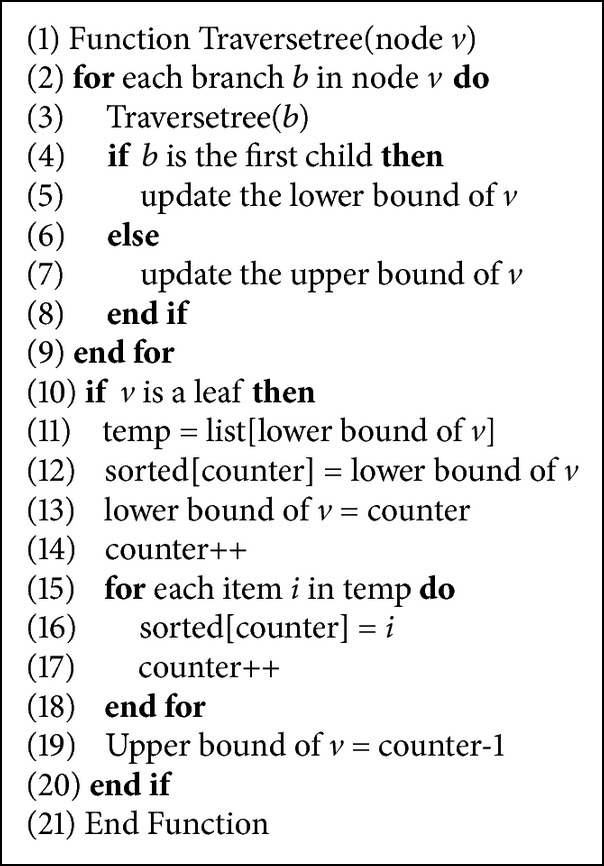
Constructing the tree without sorting the sequences.

**Algorithm 3:**
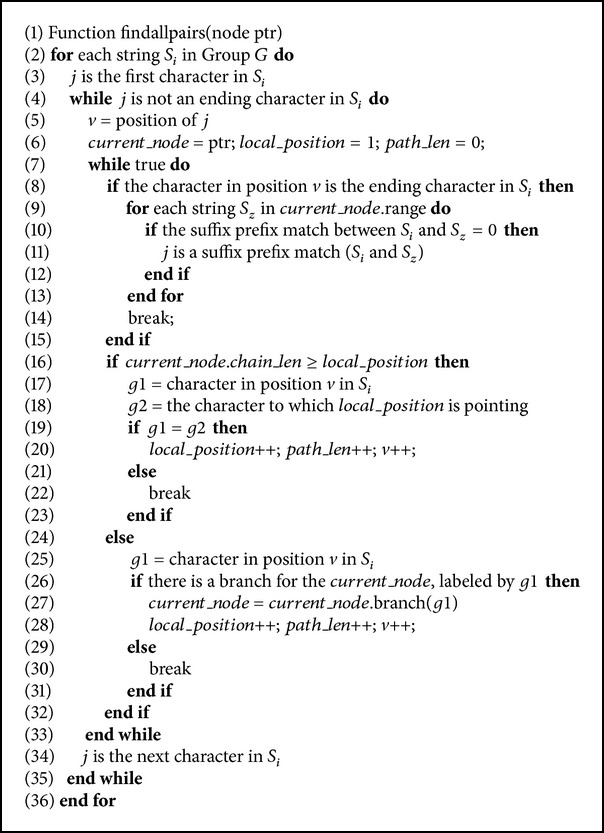
Finding all-pairs suffix-prefix.

**Algorithm 4:**
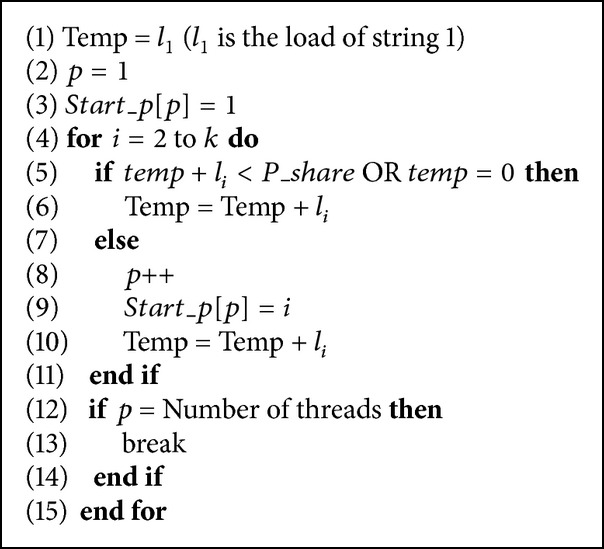
Pseudocode for the parallel algorithm.

**Table 1:** Previously presented solutions for APSP.

Solutions	Reference
Suffix tree	http://www.uni-ulm.de/fileadmin/website_uni_ulm/iui.inst.190/Forschung/Projekte/seqana/ all_pairs_suffix_prefix_problem.tar.gz

Enhanced suffix array	http://www.uni-ulm.de/fileadmin/website_uni_ulm/iui.inst.190/Forschung/Projekte/seqana/ all_pairs_suffix_prefix_problem.tar.gz

Sadakane suffix tree	http://confluence.qu.edu.qa/download/attachments/9240580/SADAApsp.zip

SGA	http://github.com/jts/sga/zipball/master

Readjoiner	http://www.zbh.uni-hamburg.de/?id=349

**Table 2:** Data sets used in experiments.

Data set	Size	Number of strings
Generated randomly using a uniform distribution	10 MB–50 GB	10^4^–66 × 10^7^
First fully public female human genome (SRR098909)	32.7 G	162 M
Illumina whole human genome (SRR866986)	9.8 G	53 M
A study in rat genome (ERR125766)	5 G	97 M
Homo sapiens	1.1 G	15 M
Exome (SRR500004)		
EST of *C*. *elegans *	167 MB	334,465
EST of *Citrusclementina *	104 MB	118,365
EST of *Citrussinensis *	154 MB	208,909
EST of *Citrustrifoliata *	46 MB	62,344
EST of *Attacephalotes *	278 MB	2,835

**Table 3:** Space consumption for Readjoiner and SOF. Clearly with Readjoner, the space consumption increases when using a larger number of threads. In addition, Readjoiner was not able to utilize all 16 threads except in two cases.

Data set	Readjoiner	Readjoiner	Readjoiner	SOF
	1 thread	4 threads	Max # of threads	1-4-16 threads
*Citrusclementina *	25 MB	57 MB	72 MB, 9 threads	52 MB
*Citrussinensis *	88 MB	107 MB	120 MB, 10 threads	80 MB
*Citrustrifoliata *	26.5 MB	39 MB	40 MB, 7 threads	25 MB
*C*. *elegans *	40 MB	48 MB	57 MB, 16 threads	100 MB
*Attacephalotes *	22.7 MB	67 MB	119 MB, 16 threads	110 MB

**Table 4:** Time consumption (in seconds) for SOF and Readjoiner using 1, 4, and 16 threads (or the maximum number of threads for Readjoiner).

Data set	1 thread	4 threads	16 threads	1 thread	4 threads	16 threads
	SOF	SOF	SOF	RJ	RJ	RJ
*Citrusclementina *	48	15	6	31	22	19
*Citrussinensis *	82	23	8	62	45	42
*Citrustrifoliata *	33	9	3	10	7	6
*C*. *elegans *	56	16	12	61	34	28
*Attacephalotes *	48	44	44	5.7	2.7	2

**Table 5:** Running SOF with large random data set using 16-core AWS machine. Number of strings in millions.

Data set	Total size	Number of strings	Time	Space
			(Minutes)	
Random	10 GB	100 M	30	15 GB
Random	20 GB	200 M	41	31 GB
Random	30 GB	300 M	76	46 GB
Random	50 GB	660 M	110	96 GB
SRR500004	1.1 GB	15 M	3	2.2 GB
ERR125766	5 GB	97 M	11	12 GB
SRR866986	10 GB	53 M	12	10 GB
SRR098909	32 GB	162 M	119	31.2 GB
